# Self-Reported Impaired Wound Healing in Young Adults and Their Susceptibility to Experiencing Immune-Related Complaints

**DOI:** 10.3390/jcm11040980

**Published:** 2022-02-13

**Authors:** Jessica Balikji, Maarten M. Hoogbergen, Johan Garssen, Joris C. Verster

**Affiliations:** 1 Division of Pharmacology, Utrecht Institute for Pharmaceutical Sciences, Utrecht University, 3584 CG Utrecht, The Netherlands; jessicabalikji@gmail.com (J.B.); j.garssen@uu.nl (J.G.); 2 Division of Plastic Surgery, Catharina Ziekenhuis, Michelangelolaan 2, 5623 EJ Eindhoven, The Netherlands; dr.mmhoog@gmail.com; 3 Global Centre of Excellence Immunology, Nutricia Danone Research, 3584 CT Utrecht, The Netherlands; 4 Centre for Human Psychopharmacology, Swinburne University, Melbourne, VIC 3122, Australia

**Keywords:** wound infection, slow healing wounds, immune functioning, immune fitness, headache

## Abstract

The current study examined to what extent individuals with wound infection (WI group), slow healing wounds (SHW group), or both (COMBI group) report poorer immune fitness and whether they experience immune-related complaints more often as compared to healthy participants (control group). Survey data from 3613 Dutch students was re-analyzed. Compared to the control group, perceived immune fitness was significantly lower by the SHW group (*p* < 0.001) and the COMBI group (*p* < 0.001), but no difference was found for the WI group (*p* = 0.059). Also, perceived immune fitness of the COMBI group was significantly worse compared to the WI group (*p* = 0.040). Compared to the control group, reduced immune fitness was reported to be significantly more frequently by the SHW group (*p* < 0.001) and the COMBI group (*p* < 0.001). Reduced immune fitness was significantly more common for the COMBI group compared to the SHW group (*p* = 0.011) and WI group (*p* = 0.001). Immune-related complaints such as headache, runny nose, coughing, sore throat, diarrhea, flu, and fever were significantly more frequently reported by individuals with impaired wound healing. The effects were most pronounced in the COMBI group, followed by the SHW group and a lesser extent the WI group. A highly significant correlation was found between perceived immune fitness and the percentage of individuals that reported impaired wound healing. In conclusion, the findings confirm that poorer immune functioning is characteristic for individuals with impaired wound healing. In follow-up studies, immune biomarkers analyses are needed to support patient-reported outcome measures.

## 1. Introduction

Chronic wounds are prevalent and constitute an underestimated public health problem [1,2]. In the United States, approximately 8.2 million adults are diagnosed with chronic wounds with or without infection, and the financial cost for chronic wound treatments have been estimated to range from $28 to $31 billion [3]. Non-healing wounds are more than just a cost burden, as they have been shown to cause loss of mobility and ability to perform daily tasks, work loss, and impaired quality of life [4].

Chronic wounds are defined as wounds that fail to progress or respond to treatment exceeding the normal expected healing time frame [5]. The most prevalent forms of chronic wounds are leg ulcers caused by chronic venous insufficiency accounting for 70–90% of ulcers found on the lower leg, followed by diabetic foot ulcers [1,6].

Wound healing progresses through three overlapping phases: acute inflammation, proliferation and granulation tissue formation, and tissue remodeling [7,8]. The immune system is heavily involved in every stage of wound healing [9]. Crucial immune cells in mediating the inflammatory phase are neutrophils and macrophages, which together eliminate necrotic tissue, debris, and bacteria from the wound [10]. Macrophages then function as the dominant cell of this stage and release several growth factors and cytokines. Thereafter, fibroblasts proliferate to function as the prominent cell of the proliferative stage. They produce collagen, which foresee wound structure and replace the fibronectin-fibrin matrix. Angiogenesis of new capillaries presents to maintain the fibroblast proliferation. In the remodeling stage, collagen synthesis and degradation achieve equilibrium. Fibroblasts arrange and cross-link the collagen, wound strength gradually grow, wound contraction occurs, and capillary and fibroblast density reduce.

Dysregulation of immune signals might lead to impaired wound healing [9]. Research showed that the wound healing in chronic wounds is arrested in a chronic inflammatory phase [9,11]. Persistent inflammation in chronic wounds is characterized by various features. Specifically, there is an excessive amount of pro-inflammatory macrophages, whereas the quantity of anti-inflammatory phenotypes is low [12]. Moreover, macrophages are less capable to clear dead neutrophils. Consequently, a highly inflammatory milieu is released with a surplus of inflammatory mediators, such as tumor necrosis factor-α (TNF-α) and interleukin-1β (IL-1β). Furthermore, macrophages release several matrix metalloproteinases (MMP), namely MMP-2 and MMP-9, which degrade the extracellular matrix and inhibit the onset of the proliferative stage of wound healing. This chronic inflammatory phase sets up a bacterial biofilm [11,12]. The biofilms interact with the host immune system by activating neutrophils and pro-inflammatory macrophages, resulting in the amassment of inflammatory cytokines [12]. This leads to imbalance between inflammatory and anti-inflammatory mediators [9,12], which thereby supports the proliferation of bacteria, leading to the vicious cycle of biofilm growth and continuous inflammation [12]. As a consequence of this hyper-inflammation, the wound does not heal but forms an ulcer that can last for years [9].

Given that immune fitness plays a central role in wound healing, the aim of the present study was to evaluate the presence of other immune-related complaints among individuals with self-reported impaired wound healing (i.e., slow healing wounds and/or wound infection) and compare the outcomes with those of individuals that do not report impaired wound healing. It was hypothesized that individuals with impaired wound healing will report significantly more immune-related complaints.

## 2. Materials and Methods

For the current analysis we combined and re-evaluated data of 3613 participants of 4 surveys that were conducted among Dutch university students, aged 18 to 30 years old [13,14,15,16]. Two of the surveys were conducted via Facebook and designed by using www.surveymonkey.com [14,16] and the other two were in paper-pencil format [13,15]. The University of Groningen Psychology Ethics Committee approved the study by Van Schrojenstein Lantman et al. (Approval code: 16072-O) [14], and the study by Sulzer et al. [16] was approved by the Ethics Committee of the Faculty of Social and Behavioral Sciences of Utrecht University granted ethical approval (approval code FETC17-061). According to the Central Committee of Research Involving Human Subjects, The Netherlands [17], no formal ethics approval was required to conduct the other two surveys [13,15]. All participants of the four studies provided informed consent.

For the current analysis we evaluated data on perceived immune fitness and immune-related complaints. Participants indicated whether or not they had experienced wound infection (WI) or slow healing wounds (SHW) during the past year. Based on their answer, participants were allocated to one of four groups, including (1) a control group (no WI or SHW), (2) a WI group, (3) an SHW group, or (4) a COMBI group (both WI and SHW). The assessment of perceived immune fitness was conducted with a one-item scale ranging from very poor (0) to excellent (10) [18]. In addition, participants reported whether they experienced reduced immune fitness at the moment of completion of the survey (yes/no question). The past year’s frequency of occurrence of immune-related complaints was assessed with a modified version of the immune fitness questionnaire [19]. Sixteen items could be scored on 5-point Likert scales (0 = never, 1 = once or twice, 2 = occasionally, 3 = regularly, and 4 = frequently) and included sore throat, headaches, flu, runny nose, coughing, cold sores, mild fever, sudden high fever, warts, pneumonia, bronchitis, sinusitis, meningitis, ear infection, diarrhea, and eye infection. Sex, age, weight, and height were demographic variables reported to further characterize the sample.

Statistical analyses were conducted with SPSS (IBM Corp. Released 2013. IBM SPSS Statistics for Windows, Version 27.0. Armonk, NY, USA: IBM Corp.). Mean and standard deviation (SD) were computed for each variable. The distribution of each variable was checked for normality by visual inspection and using the Kolmogorov–Smirnov test. This revealed that the data were usually not normally distributed, and therefore nonparametric tests were conducted. Comparisons between the four groups (control, SHW, WI, and COMBI) were conducted with the Kruskal-Wallis test. A Bonferroni’s correction was applied to account for multiple comparisons, and differences between the groups were considered significant if the adjusted *p*-value was <0.05. For data expressed as percentages, the groups were compared with the Chi-Square test, and applying a Bonferroni’s correction for multiple comparisons, differences between groups observed with the Chi-Square tests were considered statistically significant if *p* < 0.0083.

## 3. Results

Data from N = 3613 subjects (74.7% women) was included for the analysis. Demographics of the subjects are summarized in Table 1.

Women were significantly more presented in the COMBI group compared to the control group and WI group. Other differences between the groups were not statistically significant. Perceived immune fitness of the four groups is summarized in Table 2.

Compared to the control group, perceived immune fitness was rated significantly lower by the SHW group (*p* < 0.001) and the COMBI group (*p* < 0.001), but no significant difference was found for the WI group (*p* = 0.059). Also, perceived immune fitness of the COMBI group was rated as significantly worse compared to the WI group (*p* = 0.040). Other paired comparisons were not statistically significant.

Compared to the control group, reduced immune fitness was reported significantly more frequent by the SHW group (*p* < 0.001) and the COMBI group (*p* < 0.001). Reduced immune fitness was significantly more common for the COMBI group compared to the SHW group (*p* = 0.011) and WI group (*p* = 0.001).

**Figure 1:**
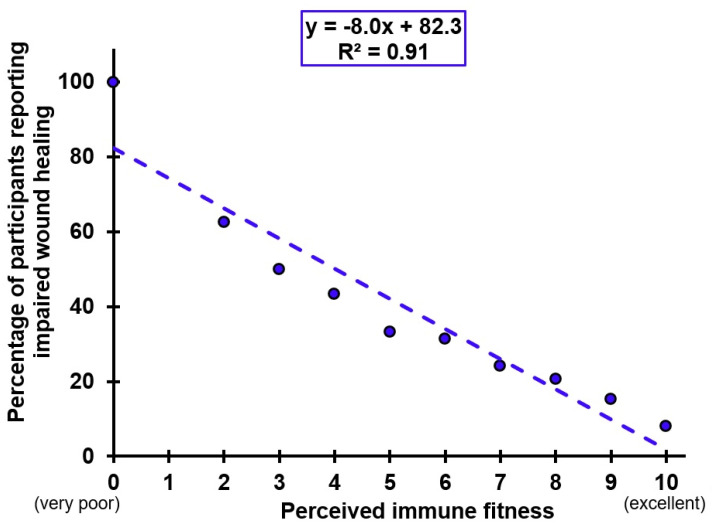
Relationship between perceived immune fitness and impaired wound healing.

Figure 1 shows the percentage of participants that reported impaired wound healing (WI, SHW, or both) in relation to their perceived immune fitness score. The percentage of participants reporting impaired wound healing was computed for each perceived immune fitness score, as follows: (the number of respondents with an immune fitness score of X that reported impaired wound healing/the total number of individuals with immune fitness score of X) × 100. The trendline describing this significant and negative relationship (y = −8.0x + 82.3, R^2^ = 0.91, *p* < 0.001) shows that with lower perceived immune ratings, more participants report impaired wound healing.

The frequency of occurrence of immune-related complaints among the four groups is summarized in Table 3. Several significant differences were found between the groups with regard to experiencing immune-related complaints (See Table 2). Most frequently reported were headache, runny nose, and coughing. Compared to the control group, headache was significantly more frequently experienced by the WI group (*p* = 0.030), SHW group (*p* = 0.001) and the COMBI group (*p* = 0.002). Compared to the control group, runny nose and coughing were significantly more frequently experienced by the WI group (*p* = 0.001 and *p* = 0.003, respectively), SHW group (both *p* < 0.001), and the COMBI group (both *p* < 0.001). Compared to the control group, sore throat was significantly more frequently reported by the SHW group (*p* = 0.013) and the COMBI group (*p* < 0.0001). Sinusitis was experienced more frequently by the WI group (*p* = 0.031), SHW group (*p* < 0.001) and the COMBI group (*p* < 0.001). No differences between the groups were found for meningitis.

Compared to the control group, pneumonia was more significantly frequently experienced by the WI group (*p* = 0.007), the SHW group (*p* = 0.016), and the COMBI group (*p* < 0.001). A significant difference was also found between the SHW and COMBI group (*p* = 0.029). Bronchitis was experienced more frequently by the WI group (*p* = 0.012) and the COMBI group (*p* = 0.002).

Compared to the control group, mild fever was significantly more frequently experienced by the WI group (*p* < 0.001), SHW group (*p* < 0.001), and the COMBI group (*p* < 0.001). Similarly, sudden high fever was experienced more frequently by the WI group (*p* = 0.001), SHW group (*p* = 0.001) and the COMBI group (*p* < 0.001). The difference between the WI group and SHW group was also significant (*p* = 0.001). Compared to the control group, flu was experienced more frequently by the SHW group (*p* = 0.002) and COMBI group (*p* < 0.001). Cold sores were experienced more frequently by the SHW group (*p* = 0.014) and the COMBI group (*p* < 0.001) compared to the control group, and also the difference between the WI group and COMBI group was statistically significant (*p* = 0.047). Diarrhea was experienced more frequently by the WI group (*p* = 0.022), SHW group (*p* < 0.001), and the COMBI group (*p* < 0.001) than by the control group.

Eye infection was experienced significantly more often by the COMBI group (*p* < 0.001) and the WI group (*p* < 0.001) than the control group. Significant differences were also found between the WI and SHW group (*p* = 0.001), and between the SHW and COMBI group (*p* < 0.001). For ear infection, a significant difference was found only between the control group and the COMBI group (*p* < 0.001). Finally, warts were experienced more frequently only by the COMBI group (*p* < 0.001), and a significant difference was also found between the SHW and COMBI group (*p* = 0.001).

## 4. Discussion

The current study confirms that poorer immune functioning and more often reporting immune-related complaints are characteristic for patients with chronic wounds. Individuals with impaired wound healing reported significantly poorer perceived immune fitness, and more often experienced reduced immune fitness. Immune-related complaints such as headache, runny nose, coughing, sore throat, diarrhea, flu, and fever were significantly more often experienced by individuals with impaired wound healing. The effects were most pronounced in the COMBI group, followed by the SHW group and (to a lesser extent) the WI group. Finally, a highly significant correlation (R^2^ = 0.91) was found between perceived immune fitness and the percentage of individuals that reported to have impaired wound healing. These findings strengthen the connection between immune fitness and wound healing, and support the idea that promoting a healthy immune fitness will be beneficial for wound healing. However immune biomarker analyses are needed to further evaluate underlying mechanisms.

The relationship between wound healing and immune fitness is bidirectional: through complex immune mechanisms, the influence of the skin microbiome extends to involve distant organ systems including the gut and brain and vice versa [20]. Illustrative for this so-called ‘‘gut-brain-skin axis’’, it has been demonstrated that individuals with reduced immune fitness experience poorer wound healing [21,22,23,24,25]. In this context, it is important to note that in wound infection and slow healing wounds different mechanisms play a role, that may explain observed differences between the two groups [26]. Also, a disturbance of the gut-brain-skin axis may have an impact on chronic inflammation that is different from acute inflammation in wound infection [27]. Alternatively, individuals with impaired wound healing could also be more susceptible to experience other immune-related diseases. There is research demonstrating that having immune-related comorbid disorders next to impaired wound healing is common. The underlying immune-related pathology of these comorbid disorders, such as diabetes [28] and irritable bowel syndrome [29], may explain their co-occurrence. However, to the best of our knowledge, there is no research published previously on the co-occurrence of more common day-to-day immune-related complaints, such as common cold, headaches, flu, or sinusitis. These complaints can also be present in relatively healthy individuals that are not being diagnosed with a (comorbid) disorder. The presence of these immune-related complaints does however point at having a poorer immune fitness (i.e., a weakened immune system), which thus can interfere with (and negatively impact) wound healing. These findings suggest that preventive measures to improve immune fitness of patients with impaired wound healing, such as dietary interventions [30], increasing sports activities [31], and improving mental wellbeing [32,33] may also have a positive effect on wound healing, and reduce the patient’s susceptibility to experiencing other immune-related complaints.

It is important to consider several potential limitations of the current study. Firstly, although the study comprised a large sample, these were all students aged 18 to 30 years old. It is therefore unclear to what extent the findings can be generalized to older age groups and nonstudents. Despite the fact that the current sample was relatively healthy and young, significant differences were found between individuals that reported impaired wound healing and the control group. It can be expected that these differences are more pronounced for patients that are actually diagnosed for having chronic wounds, and in particular when these are related to comorbid diseases such as diabetes that also have an immune-related pathology. Further research in patient groups is therefore warranted. Secondly, all data were self-reported and recall bias may have influenced reporting. Future prospective studies should therefore confirm our findings. A formal diagnosis of impaired wound healing or immune-related complaints would strengthen the study design. Thirdly, no data were collected about the underlying causes of wound infection or slow healing wounds, nor did we collect information on the type of wound, its location and size, or the duration of the healing process. This is important information that should be collected in future studies. While the current analysis demonstrates a robust association between self-reported impaired wound healing and susceptibility to immune-related complaints, the findings should be confirmed in diagnosed patients, and the impact of factors such as underlying causes and/or disease, wound type, its location and size, and the duration of the healing process should be evaluated. Finally, in the current study, perceived immune fitness was assessed. Future studies should also include biomarkers of immune functioning to support such patient-reported outcome measures.

## 5. Conclusions

Notwithstanding the study’s limitations, the results consistently show that poorer immune fitness and more frequently experiencing immune-related complaints are characteristic for individuals that report impaired wound healing.

## Figures and Tables

**Table 1:** Demographics.

Demographics	Control Group	WI Group	SHW Group	COMBI Group
N	2816	173	447	177
Sex (m/f) (%)	26.0/74.0	31.2/68.8	22.4/77.6	15.9/84.1 *^γ^
Age (years)	21.4 (2.4)	21.2 (2.5)	21.1 (2.2)	21.2 (2.5)
Weight (kg)	67.7 (11.7)	69.5 (12.5)	67.1 (11.4)	66.5 (12.2)
Height (m)	1.74 (0.1)	1.75 (0.1)	1.73 (0.1)	1.73 (0.1)
BMI (kg/m^2^)	22.3 (3.1)	22.7 (3.0)	22.3 (3.0)	22.2 (3.3)

Significant comparisons with the control group (adjusted *p* < 0.05) are indicated by *; significant differences (adjusted *p* < 0.05) between the WI and COMBI groups are indicated by ^γ^. No significant differences were found between the SHW and WI groups, and between the SHW and COMBI groups. Abbreviations: SHW, slow healing wounds; WI, wound infection; COMBI, combination of slow healing wounds and wound infection; BMI, body mass index.

**Table 2:** Perceived immune fitness.

	Control Group	WI Group	SHW Group	COMBI Group
Perceived immune fitness	7.74 (1.3)	7.50 (1.2)	7.16 (1.4) *	6.96 (1.6) *^γ^
Reduced immune fitness (%)	26.6%	32.5%	38.8% *	50.0% *^γ†^

Mean, SD, and percentage ‘yes’ are presented. Significant comparisons with the control group (*p* < 0.05) are indicated by *; significant differences (*p* < 0.05) between the SHW and COMBI groups are indicated by ^†^; significant differences (*p* < 0.05) between the WI and COMBI groups are indicated by ^γ^; no significant differences were found between the SHW and WI groups.

**Table 3:** Immune-related complaints.

	Control Group	WI Group	SHW Group	COMBI Group
Headache	1.91 (1.0)	2.13 (1.0) *	2.11 (1.0) *	2.19 (1.0) *
Runny nose	1.79 (1.0)	2.07 (1.0) *	2.02 (1.0) *	2.11 (1.0) *
Coughing	1.49 (0.9)	1.78 (0.9) *	1.76 (1.0) *	1.82 (1.0) *
Sore throat	1.36 (0.8)	1.50 (0.8)	1.49 (0.9) *	1.67 (0.9) *
Diarrhea	1.10 (0.9)	1.28 (0.8) *	1.36 (1.0) *	1.51 (1.0) *
Flu	0.65 (0.7)	0.75 (0.7)	0.81 (0.7) *	0.91 (0.7) *
Mild fever	0.57 (0.6)	0.76 (0.6) *	0.76 (0.7) *	0.84 (0.8) *
Sinusitis	0.21 (0.5)	0.32 (0.6) *	0.34 (0.7) *	0.38 (0.7) *
Cold sores	0.21 (0.6)	0.30 (0.7)	0.31 (0.7) *	0.42 (0.7) *^γ^
Ear infection	0.18 (0.5)	0.28 (0.6)	0.25 (0.6)	0.32 (0.6) *
Warts	0.16 (0.5)	0.20 (0.5)	0.20 (0.6)	0.32 (0.7) *^†^
Eye infection	0.12 (0.4)	0.26 (0.5) *	0.17 (0.5) ^‡^	0.35 (0.6) *^†^
Sudden high fever	0.10 (0.3)	0.19 (0.4) *	0.17 (0.4) *^‡^	0.28 (0.6) *
Bronchitis	0.09 (0.4)	0.21 (0.7) *	0.12 (0.5)	0.22 (0.7) *
Pneumonia	0.04 (0.2)	0.10 (0.4) *	0.09 (0.4) *	0.14 (0.4) *^†^
Meningitis	0.004 (0.07)	0.006 (0.08)	0.010 (0.10)	0.006 (0.10)

Mean and SD are presented. Significant comparisons with the control group (*p* < 0.05) are indicated by *; significant differences (*p* < 0.05) between the SHW and COMBI groups are indicated by ^†^; significant differences (*p* < 0.05) between the WI and COMBI groups are indicated by ^γ^; significant differences (*p* < 0.05) between the SHW and WI groups are indicated by ^‡^. Abbreviations: SHW, slow healing wounds; WI, wound infection; COMBI, combination of slow healing wounds and wound infection.

## Data Availability

The data is available upon request from the corresponding author.
